# Functional assessment of cancer therapy questionnaire for melanoma in the Serbian population: A factor analytic approach

**DOI:** 10.1371/journal.pone.0253937

**Published:** 2021-06-30

**Authors:** Suzana Matkovic, Jelena Dotlic, Tatjana Gazibara, Gorica Maric, Vladimir Nikolic, Natasa Maksimovic

**Affiliations:** 1 Institute of Oncology and Radiology of Serbia, Clinical Center of Serbia, Belgrade, Serbia; 2 Clinic for Obstetrics and Gynecology, Clinical Center of Serbia, University of Belgrade, Belgrade, Serbia; 3 Faculty of Medicine, University of Belgrade, Belgrade, Serbia; 4 Faculty of Medicine, Institute of Epidemiology, University of Belgrade, Belgrade, Serbia; Tabriz University of Medical Sciences, ISLAMIC REPUBLIC OF IRAN

## Abstract

The aim of this study was to examine the psychometric properties of the Functional Assessment Cancer Therapy—Melanoma (FACT-M) questionnaire in the Serbian language. The FACT-M was translated into Serbian using the standard methodology after obtaining the licence from the Functional Assessment of Chronic Illness Therapy (FACIT) translation project team. This version of FACT-M was distributed to a cohort of consecutive patients with histologically confirmed high-risk skin melanoma treated at the tertiary referral center. To examine construct validity of the FACT-M in Serbian, we performed exploratory factor analysis (EFA) and confirmatory factor analysis (CFA). The FACT-General (FACT-G) did not fit the original 4-domain structure. Instead, we accepted a 7-domain structure which, aside from physical, emotional, social and functional well-being, had domains of ‘friends’ support’, ‘illness acceptance’ and ‘fear of death’. Melanoma scale (MS) and Melanoma surgery scale (MSS) did not fit the original one-dimensional structure. The MS was observed to have 4 domains: ‘pain’, ‘skin problems’, ‘abdominal metastases’ and ‘other problems’. The MSS was observed to have 2 domains: ‘having symptoms’ and ‘no symptoms’. It is suggested that the FACT-M questionnaire is analyzed using the newly extracted domains to examine quality of life of people with high-risk melanoma in Serbia.

## Introduction

While melanoma accounts for 2–4% of skin tumors, it has the highest mortality rate [1]. Over the recent decades, the incidence of skin melanoma has been increasing; however, mortality rates do not seem to follow this trend, most likely due to early detection [2,3]. According to the GLOBOCAN projections, skin melanoma was ranked 12^th^ most common cancer in 2020 in the Republic of Serbia with incidence rates of 8.7/100,000 for men and 7.2/100,000 for women [4].

The primary treatment of melanoma involves a surgical excision. Depending on the histological findings and melanoma stage, many patients undergo additional surgical treatment and sentinel lymph node biopsy (SLNB) with or without the lymphatic basin dissection. The five-year survival rate for localized melanomas (stage 0, I and stage IIA according to the American Joint Committee on Cancer [AJCC-TNM] TNM classification) is 98.4% [5,6]. Patients with high-risk skin melanomas, stages IIB to IV, are more likely to have a worse prognosis as the rate of recurrence is 35–40% higher than that in earlier stages [7] and their overall five-year survival rate is less than 60% [6]. Therefore, people with high-risk melanoma represent a subgroup of all melanoma patients, who are more likely to have poorer disease outcomes.

Because of the increased risk of recurrence in people with high-risk melanoma, these individuals suffer considerable psychological distress and are likely to experience an impaired quality of life (HRQoL) compared to their gender and age-matched counterparts [8]. Moreover, the treatment and risk of recurrence may cause additional strain and prolong the recovery. A previous study reported that people with melanoma have worse HRQoL at the time of diagnosis and at the beginning of follow-up [9]. Exploration of HRQoL among persons with melanoma could pinpoint specific issues that these individuals encounter and may help to improve the treatment and disease outcomes. To achieve this, it is necessary to administer HRQoL questionnaires specific to melanoma. However, thus far, no validated instruments for HRQoL measurement have been used in the Serbian language.

The aim of this study was to examine psychometric properties of the Functional Assessment Cancer Therapy—Melanoma (FACT-M) questionnaire in the Serbian language.

## Material and methods

### Participants

This study included all consecutive patients with histo-pathologically confirmed high-risk skin melanoma treated at the tertiary referral center Institute of Oncology and Radiology of Serbia (IORS) from June 2017 until December 2018. Based on the cancer register in Serbia in 2015, a total of 456 people were diagnosed with melanoma [10]. The incidence of melanoma patients in Serbia corresponds with populations of similar size. Out of all diagnosed melanomas in the population of Serbia high-risk melanomas account for around 40% [10]. The IORS is one of the three referral centers in Serbia where high-risk melanomas are treated, covering mostly the central parts of the country. The majority of melanoma patients are diagnosed and treated in our institution, which makes our study sample representative. To estimate the sample size according to Slovin’s formula, we used the average annual number of high-risk melanoma patients operated on in IORS (91.36) and a margin of error of 0.05. The calculated sample size was 74 patients. With this sample the post-hoc study power for the whole FACT-M was 100%, while it was 79% for the MSS subscale, when compared with the original study [13].

All patients were informed of the study objectives and provided signed informed consent. The study was approved by the Ethics Committee of the Institute for Oncology and Radiology of Serbia (approval no. 1413–01).

The inclusion criteria were histo-pathologically confirmed high-risk skin melanoma (stages IIB, IIC, III and IV), aged over 18 and speaking the Serbian language. The exclusion criteria were having a cognitive disorder or a mental disability that could interfere with the process of completion of the questionnaire and refusal to participate.

### Data collection

The socio-demographic data were collected through interview after the primary surgical treatment (excision of the tumor, sentinel lymph node biopsy with or without a complete lymph node dissection), while data about melanoma were collected from the medical records. The following socio-demographic data were obtained: gender, age, marital status (single, married/couples, widow or divorced), level of education (none, elementary, secondary, high—university level) and occupation (unemployed, farmer, housewife, employed, retired).

The following data about melanoma were collected: disease stage according to the AJCC-TNM TNM classification, metastatic disease (no or yes), initial surgical treatment (yes or no), radiotherapy (yes or no) and systemic therapy i.e. immunotherapy, chemotherapy, etc. (yes or no).

All patients responded to two questionnaires: The Medical Outcomes Study Short Form 36 version 2 questionnaire (SF-36v2) [11] and the disease-specific FACT-M questionnaire. The SF-36v2 is a widely used generic HRQoL instrument containing 36 questions and has been approved for use in the Serbian language. The SF36v2 relates to the four weeks preceding the survey and addresses eight HRQoL domains: Physical Functioning (PF), Role-Physical (RP), Bodily Pain (BP), General Health (GH), Vitality (VT), Social Functioning (SF), Role-Emotional (RE), and Mental Health (MH). The former 4 domains are combined and their mean value represented physical composite score (PCS) and the mean of the latter 4 domains represented mental composite score (MCS). The total SF-36v2 score is obtained as the average of the two composite scores. The SF-36v2 was scored using the Quality Metric’s Health Outcomes^™^ Scoring Software 5.1.

### Functional Assessment Cancer Therapy—Melanoma

The FACT-M is composed of the Functional Assessment of Cancer Therapy-General (FACT-G) [12] (so-called core component) and melanoma-specific modules. Therefore, the FACT-M is composed of 3 separate subscales: 1) FACT-G which comprises 4 domains: physical well-being (PWB), social well-being (SWB), emotional well-being (EWB), functional well-being (FWB): 2) Melanoma scale (MS) which includes 16 items related to general symptoms that can be associated with melanoma and 3) Melanoma surgery scale (MSS) includes 8 items specific for the problem at the site of the surgery. The initial psychometric testing of the FACT-M suggested that the validity (as measured by the Cronbach’s alpha coefficients) of the FACT-G was >0.7 while of the MS and the MSS were >0.8. The test-retest reliability was >0.8 for all 3 subscales [13].

Although responses for all 3 subscales range from 0 (not at all) to 4 (very much), these three subscales are, in fact, different constructs. For this reason, the FACT-M questionnaire is not structurally coherent, and therefore can be analyzed as 3 separate subscales (with 3 separate subscale scores) within the same questionnaire or through its two major summary scores [9,13]. The scoring is performed according to the official guidelines. First, the process of reverse scoring of the indicated items should be performed. Then, the calculation of the domain scores is carried out as follows: the item scores are summed up and multiplied by the number of items in that domain; subsequently, the product is divided by the number of items answered. As the subscales MS and MSS have just one domain, the domain score equals the subscale sore. The FACT-G total/summary score is calculated by summing the four domain scores that make up this subscale: PWB, SWB, EWB and FWB. Finally, the FACT-M total/summary score is obtained by adding the MS subscale score to the FACT-G total score. The Melanoma Surgery Scale is always assessed as an independent scale, and therefore, it is not included in any of the summary scores. Higher scores indicate a better quality of life.

The license to use the FACT-M questionnaire was obtained from the Functional Assessment of Chronic Illness Therapy (FACIT) Organization. The FACT-M questionnaire was translated as follows: (1) two independent investigators, native speakers of the Serbian language performed the translation from English to Serbian; (2) two different versions were reconciled by a third investigator who was not involved in the first step; (3) the fourth translator, a native English speaker, who was also fluent in the Serbian language, performed the back translation of the reconciled version; (4) fourth step: a language coordinator together with a clinical expert team made the final version of the FACT-M in Serbian. All five versions of the questionnaire were submitted to the FACIT Organization.

The pre-final Serbian version of the FACT-M questionnaire was tested on 10 patients diagnosed with high-risk melanoma at the IORS. The goal of the pilot testing was to determine whether any items were difficult to understand or culturally irrelevant. The patients completed the pre-final version of the questionnaire after which they answered questions from the cognitive debriefing script as prepared by the FACIT.

All patients included in the pilot testing were interviewed by the investigator. All comments made by patients were recorded. The patients indicated that there were no questions that were incomprehensible, abusive, irrelevant or disturbing. They stated that the questions were comprehensive. Patients made no suggestions to include additional questions. The data obtained during the pilot testing was submitted, and the approval for the final Serbian questionnaire version was obtained from the FACIT Organization. The final Serbian FACT-M (Version 4) was approved, following a statistical confirmation by the FACIT translations project team.

### Statistical analysis

The descriptive and analytical statistical methods were used. The continuous variables were shown as mean ± SD and the categorical variables were presented as proportions. The internal consistency of the Serbian version of the FACT-M scale was assessed by the Cronbach’s alpha coefficient and McDonald omega coefficient. The level of > 0.7 for both coefficients was deemed adequate [14]. McDonald’s coefficient was calculated using the JASP software.

To examine the construct validity of the FACT-M in Serbian we performed exploratory factor analysis (EFA) (in the SPSS version 20.0) and the confirmatory factor analysis (CFA) (using the AMOS software version 18.0).

In the EFA the Varimax rotation was chosen for the orthogonal approximation of the factor structure. We based the observations upon the eigenvalues higher than 1.0, which suggested the number of factors in the construct of each FACT-M subscale. Factor loadings were analysed within the rotated component matrix. The values of factor loadings were grouped according to similarities starting from the highest observed values in each factor. Communality indices of > 0.4 are considered appropriate [15].

To examine the constructs of the FACT-M in Serbian in CFA we assessed the goodness-of-fit of the model by χ^2^ statistics (χ^2^/df), comparative fit index (CFI), adjusted goodness of fit index (AGFI), and root mean square error of approximation (RMSEA). Values of χ^2^/df (CMIN/DF) below 5.0, RMSEA below.08 together with CFI and AGFI above.90 indicated good model fit [16].

The concurrent validity of the FACT-M was assessed by correlating the FACT-M scores with the domain and composite scores of the SF-36v2 (Pearson correlation) and the intercorrelations between FACT-M total and various domain scores were also evaluated.

## Results

### Description of the study sample

The study included a total of 81 patients with high-risk melanoma. Basic demographic and clinical characteristics of patients are presented in Table 1. Investigated patients were predominantly male (59.8%), married (67.8%), with a secondary level of education (65.5%) and employed (51.6%). The average age was 57.8±13.1 years. The majority of the participants (35.6%) were in IIC disease stage and had ECOG PS grade 0 (82.8%). At the time of investigation only 4.6% patients had metastatic disease. All patients were surgically treated, while in 5.7% of patients a systemic therapy was also applied.

**Table 1:** Demographic and clinical characteristics of the participants.

Variable	n (%)
Gender	
Male	52 (59.8)
Female	35 (40.2)
Age (years)*	57.8±13.1
Marital Status	
Single	10 (11.5)
Married	59 (67.8)
Widow	8 (9.2)
Divorced	9 (10.3)
Free union	1 (1.1)
Level of Education	
None	2 (2.3)
Elementary (up to 8 years)	11 (12.6)
Secondary (up to 12 years)	57 (65.5)
High—university (≥12 years)	17 (19.5)
Occupation	
Unemployed	8 (9.2)
Farmer	5 (5.7)
Housewife	5 (5.7)
Employed	45 (51.6)
Retired	24 (27.6)
Disease stage	
IIC	31 (35.6)
IIIA	13 (14.9)
IIIB	12 (13.8)
IIIC	25 (28.7)
IV	6 (6.9)
Metastatic disease	
No	83 (95.4)
Yes	4 (4.6)
Initial surgical treatment	
Yes	87 (100.0)
No	0 (0.0)
Radiotherapy	
Yes	0 (0.0)
No	87 (100.0)
Systemic therapy (immunotherapy, chemotherapy, etc.)	
Yes	5 (5.7)
No	82 (94.3)

*values are denoted as mean±sd.

### Internal consistency

The mean, range and standard deviations for scores in all 6 subscales are shown in Table 2. We observed that responses for the item labeled as Hep3 (“I have had fevers”) pertaining to the MS subscale were all 0 (i.e. none of the participants reported having had fevers). However, because the item Hep3 needs to be transformed, as per scoring guidelines, prior to calculation of the MS subscale score, the value 4 was assigned to all values 0. This means that we were able to calculate the MS score with all the items including transformed Hep3. Still, because no variability was observed for this item, it was not possible to conduct a meaningful analysis of the internal consistency and construct of the subscale when item Hep3 was part of the MS subscale. Therefore, this item was removed and the MS subscale was considered as a 15-item instead of a 16-item subscale for further analysis.

**Table 2:** Descriptive statistics and reliability of the FACT-M questionnaire.

Subscale	Mean (range)	Standard deviation	Cronbach’s alpha	McDonald’s omega
PWB (7 items)	24.5 (12–28)	3.6	0.818	0.815
SWB (7 items)	22.9 (12–29)	4.0	0.792	0.811
EWB (6 items)	19.1 (10–24)	4.4	0.566	0.776
FWB (7 items)	20.4 (10–28)	4.6	0.832	0.822
FACT-G total	86.9 (53–106)	11.6	0.876	0.896
MS (16 items)	56.9 (37–64)	5.3	0.676	0.748
FACT-M total	143.8 (101–170)	15.9	0.912	0.904
MSS (8 items)	28.3 (12–32)	3.6	0.790	0.851

PWB—Physical wellbeing; SWB—Social/family wellbeing; EWB—Emotional wellbeing; FWB—Functional wellbeing; MS—Melanoma subscale; MSS—Melanoma surgery scale.

Table 2 also shows coefficients alpha and omega for four subscales of FACT-G, MS and MSS. Cronbach’s alpha coefficients was appropriate for the whole FACT-M, but lower than what is commonly acceptable for EWB and MS domains. However, McDonald’s omega coefficients were adequate for all subscales.

### Construct validity—Exploratory and confirmatory factor analysis

The results of EFA are shown in Tables 3 and 4, while results of the CFA are presented in Table 5, S1 and S2 Figs.

**Table 3:** Correlation coefficients of the FACT-G subscale items of the FACT-M and factors extracted after Varimax rotations in EFA.

Items	Factor loading							Communality index
	Original scale domains				New scale domains in Serbian FACT-G			
	PWB	SWB	EWB	FWB	Friends’ support	Illness acceptance	Fear of death	
GP1_T	0.52	0.05	0.28	0.47	-0.16	0.02	-0.08	0.52
GP2_T	0.17	0.31	0.22	0.52	-0.28	-0.01	0.17	0.34
GP3_T	0.76	-0.01	0.06	0.12	0.05	-0.02	0.25	0.57
GP4_T	0.56	0.09	0.27	0.20	0.46	0.06	0.08	0.49
GP5_T	0.78	0.20	0.04	0.11	0.07	0.25	0.11	0.47
GP6_T	0.60	0.20	0.13	0.52	0.04	0.01	0.06	0.65
GP7_T	0.19	0.12	0.68	0.21	0.19	-0.31	-0.10	0.25
GS1	0.07	0.25	0.06	0.12	0.80	0.11	0.01	0.44
GS2	0.12	0.80	-0.05	0.09	0.31	0.07	-0.06	0.71
GS3	0.02	0.36	-0.11	0.15	0.76	0.03	0.04	0.58
GS4	0.13	0.82	0.01	0.07	0.13	0.15	0.22	0.70
GS5	0.02	0.70	-0.03	0.12	0.26	-0.16	0.39	0.69
GS6	0.08	0.79	0.24	0.29	0.04	0.15	-0.13	0.67
GS7	-0.02	-0.14	0.50	-0.36	0.02	-0.06	0.16	0.36
GE1_T	-0.01	0.05	0.67	0.14	-0.04	0.41	0.07	0.63
GE2	0.22	-0.04	0.54	0.05	0.04	0.48	-0.27	0.47
GE3_T	0.22	0.18	0.62	0.17	0.01	0.09	0.34	0.60
GE4_T	0.13	0.08	0.11	0.06	0.13	0.01	0.78	0.46
GE5_T	0.31	0.12	0.25	0.08	-0.18	0.15	0.59	0.47
GE6_T	0.14	-0.05	0.68	-0.05	-0.15	0.18	0.39	0.68
GF1	0.27	-0.07	-0.01	0.64	0.33	-0.06	0.19	0.55
GF2	0.21	0.03	-0.05	0.76	0.28	-0.08	0.02	0.64
GF3	0.23	0.22	-0.08	0.69	0.17	0.32	-0.05	0.71
GF4	0.09	0.09	0.06	0.02	0.02	0.79	0.02	0.31
GF5	0.05	0.11	0.18	0.40	0.22	0.54	0.28	0.44
GF6	0.12	0.20	0.06	0.65	0.15	0.44	0.13	0.65
GF7	-0.11	0.28	0.42	0.64	-0.13	0.10	-0.01	0.51

PWB—Physical wellbeing; SWB—Social/family wellbeing; EWB—Emotional wellbeing; FWB—Functional wellbeing; Shaded values were considered to belong in the observed domain; Items that are marked with “_T” denote that the raw values were transformed (i.e. inversed). The coding of items in the original questionnaire available on http://www.facit.org denotes those items expressed as GP_number belong to the PWB; items expressed GS_number belong to the SWB; items expressed as GE_number belong to the EWB; items expressed as GF_number belong to the FWB. Full items according to codes are available in the Supplemental Material.

**Table 4:** Correlation coefficients of the Melanoma scale and Melanoma surgery scale items of the FACT-M and factors extracted after Varimax rotations in EFA.

Melanoma scale items	Extracted domains				Communality index
	Pain	Skin problems	Abdominal metastases	Other problems	
M1_T	0.75	-0.17	-0.02	0.17	0.10
M2_T	0.32	0.69	0.10	-0.08	0.06
M3_T	-0.10	0.82	-0.08	0.14	0.12
B1_T	0.25	-0.07	0.28	0.63	0.40
IUT4_T	0.18	0.06	-0.06	0.78	0.59
An10_T	0.71	0.24	-0.04	0.02	0.10
C1_T	-0.03	0.10	0.78	0.11	0.02
C6	0.18	0.01	0.70	-0.06	0.01
M5_T	0.42	0.01	0.20	0.57	0.43
M6_T	-0.17	-0.08	0.70	-0.07	0.01
ITU3_T	-0.02	-0.02	-0.18	0.71	0.38
MS8_T	-0.06	0.18	-0.03	0.56	0.29
M8_T	-0.21	0.51	0.08	0.52	0.33
M9_T	0.01	-0.03	-0.01	0.55	0.23
HI7_T	0.23	0.37	-0.01	0.66	0.61
Melanoma surgery scale items	Extracted domain				Communality index
	Symptoms yes		Symptoms no		
M10_T	0.42		0.66		0.51
M11_T	0.52		0.73		0.70
M12_T	0.52		0.59		0.57
M13_T	0.71		0.16		0.46
M14_T	0.85		0.29		0.77
M15_T	0.82		0.07		0.52
M16_T	0.67		0.07		0.36
M17	-0.21		0.72		0.04

Values in shade were considered to belong in the observed domain; Items that are marked with “_T” denote that the raw values were transformed (i.e. inversed). The coding of items in the original questionnaire available on http://www.facit.org denotes those items coded from M1_T to HI7_T belong to the Melanoma scale. In a similar manner, items coded from M10_T to M17 belong to the Melanoma surgery scale. Full items according to codes are available in the Supplementary Material.

**Table 5:** Results of the confirmatory factor analysis according to domains and subscales of the FACT-M questionnaire.

CFA model fit parameters	Model FACT-G original 4 factors	Model FACT-G new 7 factors	Melanoma scale new 4 factor model	Melanoma surgery scale new 2 factor model
p	0.001	0.001	0.102	0.125
CMIN/DF	10.665	10.659	10.217	10.378
GFI	0.695	0.716	0.869	0.929
AGFI	0.634	0.864	0.809	0.865
NFI	0.597	0.816	0.705	0.920
CFI	0.779	0.791	0.924	0.976
PCFI	0.700	0.779	0.731	0.662
RMSEA	0.091	0.091	0.052	0.069

p: Significance level; CMIN/DF: Minimum discrepancy divided by its degrees of freedom; GFI: Goodness-of-fit index; AGFI: Adjusted GFI; NFI: Normed fit index; CFI: Comparative fit index; PCFI: Parsimony-adjusted CFI; RMSEA: Root mean square error of approximation.

After the initial running the CFA for FACT-G according to the original 4 domains, a measurement model with an acceptable fit for our population was observed. Still, this model had some parameters that were not quite adequate. The PWB showed high covariances with FWB and EWB. Moreover, when we assessed the MSS and MS according to one-domain structure, we observed that the fit indices were somewhat poor.

Because of this, we decided to perform the EFA of all item responses of all investigated patients by setting the original structure of the FACT-M (4 domains for FACT-G, 1 domain for MS and 1 domain for MSS). We observed that the 4-factor FACT-G explained 54.4% of variance, 1-factor MS explained 24.8% of variance and 1-factor MSS explained 49.7% of variance. These variances were considered low.

For this reason, we performed the EFA of all item responses from the whole sample where factors were extracted based on factor loadings. It was found that FACT-G in our population had 7 instead of 4 domains (Table 3). The total variance explained by 7 domains was 69%. Most items from the original 4 domains remained within those domains. Items GP2 (“I have nausea”) was moved from the Physical to the Functional well-being domain and item GP7 (“I am satisfied with my sex life”) was moved from the Physical to the Emotional well-being domain. However, the observed communality indices showed that items with values < 0.4 pertained to those items that were observed outside of the original domains.

We observed that the newly extracted domains in the FACT-G subscale had 2 items each and all 3 new domains could be reasonably explained. The new domain “Friends’ support” was composed of items GS1 (“I feel close to my friends) and GS3 (“I get support from my friends”), which originally belonged to the Social well-being domain. The new domain “Illness acceptance” was composed of GF4 (“I have accepted my illness”) and GF5 (“I am sleeping well”), which originally belonged to the Functional well-being domain. Finally, the new domain “Fear of death” was composed of GE4 (“I feel nervous”) and GE5 (“I worry about dying”), which belonged originally to the Emotional well-being domain.

The MS subscale, which originally had 1-factorial structure, on EFA showed 4 domains: 15 items clustered according to Pain (item M1), Skin problems (items M2, M3), Abdominal metastases (items C1, C6, M6) and Other problems (all other items). The total variance explained by the 4 domains was 56.3%. The communality indices were low overall for most items (Table 4).

The MSS subscale, which originally had 1-factorial structure, showed 2 domains on EFA. Items were distributed between domains in line with the existence of symptoms (Symptoms yes and Symptoms no). The communality indices were low for M16 and M17. The total variance explained was 64.1% (Table 4).

Finally, we once again performed the CFA for the newly acquired seven-factor FACT-G, four-factor MS and two-factor MSS subscales. We chose to perform this CFA on the whole sample as well so that we would specifically test and verify the previously observed factors. This CFA revealed that the model fit for seven-factor FACT-G observed in the Serbian population was also not ideal, similarly to the original structure, but still was somewhat better regarding its estimates (Table 5). As for the MS subscale, in order to execute the CFA two symptom i.e. items (having fever and abdominal cramps) had to be omitted due to a lack of variance in patient responses. The CFA proved that the model of the MS with four domains was an appropriate fit for our population (Table 5). Lastly, the CFA proved that the model of the MSS subscale with two domains was an appropriate fit for our population (Table 5).

### Concurrent validity

The FACT-M questionnaire in Serbian was correlated with almost all scale scores. The correlation was not observed between the MSS with SWB (p<0.05) and EWB (p>0.05) (S1 Table). Further, the domains of FACT-M were correlated with the domains, composite scores and the total score of SF-36v2 questionnaire (S2 Table). The SWB correlated significantly (p<0.05) with Physical Functioning, Role Physical, Social functioning, Pain and Physical and Mental composite scores. The EWB domain correlated (p<0.01) with all SF-36 domains except with Physical functioning (p>0.05). Finally, MSS was not correlated (p>0.05) with Role Emotional, Mental Health and General health.

## Discussion

This study assessed psychometric properties of the FACT-M questionnaire. Over the recent years, the family of FACT questionnaires has been increasingly used to investigate the HRQoL among persons who have different cancers and/or chronic illnesses. We presented the first examination of the FACT-M questionnaire in a Serbian cohort of high-risk melanoma patients.

The descriptive and clinical characteristics of our study sample are consistent with other studies exploring this health problem [12,17,18]. Our results indicate that overall internal consistency of the Serbian version of FACT-G, MS and MSS have good internal consistency. While Cronbach’s alpha coefficient is generally used to assess the internal consistency, McDonald’s omega has been increasingly used, as it is suggested to better represent this feature of the questionnaire [14].

The domains in which the highest alpha coefficients were observed are functional wellbeing and physical wellbeing. It has been similarly shown in other studies for these domains [12,19]. The same holds true for the omega coefficients [19]. Contrarily, emotional wellbeing had the lowest internal consistency. The emotional wellbeing domain also had lower Cronbach’s alpha in other studies that used FACT to explore HRQoL of patients with malignancies [17]. A possible explanation for this finding could be the fact that most investigated patients had early melanoma stages, which perhaps was not perceived as emotionally burdensome. However, the physical and social impact after having surgical intervention was more consistent. Another possible reason may be the fact that 2/3 of our study group were men. It is known that women have increased stress vulnerability, a stronger emotional reaction compared to men when confronted with traumatic events like being diagnosed with melanoma [20].

While previous studies focused on conducting the CFA and other aspects of psychometric testing, [19,21] to our knowledge there is no available data about whether FACT-M has been examined using the EFA in other populations of melanoma patients. According to factor analysis FACT-G subscale in the Serbian population could be used in further research and clinical practice with the four original domains. However, the construct with three more domains than the original version seems to fit the Serbian population somewhat better, probably due to social and cultural specificities of all or some melanoma patients in Serbia. Serbian people are generally amicable and hospitable and consequently their friendships are very important to them. This might explain why the only two items regarding relationship with friends in FACT-G formed a new factor. The remaining two new factors incorporate two different aspects of dealing with malignant illness. One group of patients with melanoma accept their illness without psychological issues, such as anxiety and depression. Therefore, their daily functioning is not significantly impaired. Contrary to this, the other group seems to be more burdened as they worry about dying. These feelings appear to be more important for Serbian melanoma patients than all other emotions. Further research on larger samples of patients with all melanoma stages should be undertaken to investigate the most appropriate factorial construct of FACT-G subscale with regards to specific interpretation and perspective of melanoma patients.

In the Serbian population, the MS and MSS subscales were found to have four and two domains, respectively. Metric evidence for these factors suggested adequacy of these constructs. Moreover, it was evident that items of the MS clustered according to organs system causing the symptoms, while items of the MSS were divided based on the symptoms presence. Therefore, it can be concluded that Serbian patients clearly understand melanoma and surgical therapy related symptoms and differentiate their origin and type. The only issue with the items of MS was the fact that two items had to be excluded from analysis due to lack of variability. In fact, none of the examined patients reported fever although it is a well known symptom of advanced malignancy. One obvious reason for this may be the fact that we did not have many stage IV patients in our sample. The circumstances that the investigated stage IV patients were all afebrile may be due to chance. Consequently, we believe that all the original items of MS should be kept in the Serbian version as well because other patient samples could easily have all of the proposed symptoms. We recommend that other authors conduct the EFA on their sets of melanoma patients to further explore the construct of FACT-G, MS and MSS across cultures.

The Social/family wellbeing domain did not correlate with the role limitations due to emotional problems, emotional wellbeing and general health domains. Also, the emotional wellbeing domain does not correlate with the physical functioning domain and the melanoma surgery scale domain does not correlate with the role limitations due to emotional problems, emotional wellbeing and general health domains. This is expected because no correlations are shown between domains which evaluate different aspects. Strong correlations are shown between the domains which assess similar or associated concepts confirming the concurrent validity of the FACT-M questionnaire. Similar correlations between FACT-M and other HRQoL questionnaires have been reported in other studies [19,22].

### Limitations and future implications

Our study has some limitations. No test-retest reliability was performed and a lack of validation studies limits the comparison of our results with different study groups. We predominantly involved patients in the no evidence of disease (NED) stage, which we would also single out as an advantage, because less research is focused on patients in the NED stage of the disease. Although we took into account the frequency of high-risk melanoma patients in our institution to calculate the sample size and despite high post-hoc study power, our study is limited by a small sample size which depends on rather low high-risk melanoma incidence rates in Serbia. Due to a small sample, further stratification of patients according to different disease stages and sub-stages or other features would decrease the statistical power, so we opted to explore these issues in further studies. Some authors consider that small sample sizes are not sufficient to conduct EFA and CFA; however, others suggest that comparative analysis of datasets which differ in size generally do not yield substantial differences in metric properties of the questionnaire [23]. Further, although greater sample size can increase the value of factor loadings, some literature data indicates that there is neither an absolute minimum nor a critical ratio of sample size to number of items [24]. In our case, item-to-respondent ratio for FACT-G was 1:3, for MS 1:5 and for MSS the ratio was 1:10.1.

Finally, adequacy of factor analysis depends not only on sample size, but on factor loadings and how items and factors logically relate to one another, as well as with the research subject. We opted to perform factor analysis as appropriate factor loadings were obtained. Although some authors recommend that EFA and CFA should be performed on separate samples, we performed both analyses on the same study sample. If questionnaire is first explored with EFA and successively by CFA in the same sample, the validity of those restrictions implied by the CFA which were not part of the EFA (e.g., fixed cross-loadings, uncorrelated errors) are being tested. If the structure is correct (the factors actually represent an existing entity and the supposed causal effects i.e. factor loadings are correctly specified), this test makes sense. In our case, no conflict between analyses was present as the CFA was applied for the second time with the exact intention to confirm the hypothesis that arose from the previously performed EFA. Future studies should analyze FACT-M in more details on larger samples and offer potential modifications of items.

## Conclusion

This study found that domains and subscales of the Serbian version of the FACT-M have good internal consistency. The two melanoma-specific subscales of the FACT-M questionnaire in the Serbian language showed overall acceptable construct validity. The FACT-G in the Serbian population of high-risk melanoma patients could be more suitable as a seven-domain construct, rather than the original four-domain construct. The FACT-M can be used to examine HRQoL among patients with high-risk skin melanoma in the Serbian language.

## Supporting information

S1 Fig CFA for HRQOL models with original (a) 4 and (b) new 7 factors. (TIF)

S2 Fig CFA for (a) symptoms and (b) surgery models (standardized coefficients). (TIF)

S1 Table Inter-subscale correlations. (DOC)

S2 Table Correlations of FACT-M domain scores with SF-36 questionnaire scores. (DOC)

S1 Appendix FACT-M questionnaire. (DOC)

S2 Appendix FACT-M Serbian data. (XLS)

## References

[1] Swetter SM. Dermatological perspectives of malignant melanoma. Surg Clin North Am. 2003;83:77–95. doi: 10.1016/s0039-6109(02)00091-9 12691451

[2] Kosary CL, Altekruse SF, Ruhl J, Lee R, Dickie L. Clinical and prognostic factors for melanoma of the skin using SEER registries: Collaborative stage data collection system, version 1 and version 2. Cancer. 2014;120:3807–14. doi: 10.1002/cncr.29050 25412392

[3] Guy GP Jr., Thomas CC, Thompson T, Watson M, Massetti GM, Richardson LC. Vital signs: Melanoma incidence and mortality trends and projections—United States, 1982–2030. MMWR Morb Mortal Wkly Rep. 2015;64:591–6. 26042651 PMC4584771

[4] Global Cancer Observatory (GLOBOCAN). Summary statistic Serbia 2020. [cited 2020 November 30]. https://gco.iarc.fr/today/data/factsheets/populations/688-serbia-fact-sheets.pdf.

[5] Gershenwald JE, Scolyer RA, Hess KR, Sondak VK, Long GV, Ross MI, et al. for members of the American Joint Committee on Cancer Melanoma Expert Panel and the International Melanoma Database and Discovery Platform. Melanoma staging: Evidence-based changes in the American Joint Committee on Cancer eighth edition cancer staging manual. CA Cancer J Clin. 2017;67:472–92. doi: 10.3322/caac.21409 29028110 PMC5978683

[6] Melanoma Research Alliance. Melanoma Survival Rates. [cited 2020 November 30]. https://www.curemelanoma.org/about-melanoma/melanoma-staging/melanoma-survival-rates/.

[7] Tarhini AA, Lorigan P, Leachman S. Operable Melanoma: Screening, Prognostication, and Adjuvant and Neoadjuvant Therapy. Am Soc Clin Oncol Educ Book. 2017;37:651–60. doi: 10.1200/EDBK_174930 28561661

[8] Barbato MT, Bakos L, Bakos RM, Prieb R, Andrade CD. Predictors of quality of life in patients with skin melanoma at the dermatology department of the Porto Alegre Teaching Hospital. Ann Bras Dermatol. 2011;86:249–56. 10.1590/s0365-05962011000200007 21603807

[9] Cormier JN, Davidson L, Xing Y, Webster K, Cella D. Measuring quality of life in patients with melanoma: development of the FACT-melanoma subscale. J Support Oncol. 2005; 3:139–45. 15796446

[10] Institute of Public Health of Serbia. Cancer incidence and mortality in Central Serbia 2015. (2017). http://www.batut.org.rs/download/publikacije/Incidencija%20i%20mortalitet%20od%20raka%202015.pdf.

[11] Ware JE, Sherbourne CD. The MOS 36-Item Short-Form Health Survey (SF-36). Med Care. 1992;30:473–83. 1593914

[12] Functional Assessment of Chronic Illness Therapy. [cited 2020 November 30]. http://www.facit.org/FACITOrg/Questionnaires.

[13] Cormier JN, Ross MI, Gershenwald JE, Lee JE, Mansfield PF, Camacho LH, et al. Prospective assessment of the reliability, validity, and sensitivity to change of the functional assessment of cancer therapy-melanoma questionnaire. Cancer. 2008;112:2249–57. doi: 10.1002/cncr.23424 18383513

[14] Dunn TJ, Baguley T, Brunsden V. From alpha to omega: a practical solution to the pervasive problem of internal consistency estimation. Br J Psychol. 2014;105:399–412. doi: 10.1111/bjop.12046 24844115

[15] Hair JF, Black WC, Babin BJ, Anderson RE. Multivariate Data Analysis: A Global Perspective. 7^th^ ed. New Jersey: Pearson Prentice Hall; 2010.

[16] Child D. The Essentials of Factor Analysis. 3^rd^ ed. New York, NY: Continuum International Publishing Group Ltd; 2006.

[17] Di Bella O, Cocchiara RA, De Luca A, Frusone F, Aceti V, Sestili S, et al. Functional Assessment of Cancer Therapy Questionnaire for Breast Cancer (FACT-B+4): Italian version validation. Clin Ter. 2018;169:e151–4. doi: 10.7417/T.2018.2071 30151547

[18] Arli SK, Gurkan A. Validity and Reliability of Turkish Version of the Functional Assessment of Cancer Therapy-Brain Questionnaire. Cancer Nurs. 2017;40:224–9. doi: 10.1097/NCC.0000000000000390 27171811

[19] Bharmal M, Nolte S, Henry-Szatkowski M, Hennessy M, Schlichting M. Update on the psychometric properties and minimal important difference (MID) thresholds of the FACT-M questionnaire for use in treatment-naïve and previously treated patients with metastatic Merkel cell carcinoma. Health Qual Life Outcomes. 2020;18:145. doi: 10.1186/s12955-020-01402-3 32430019 PMC7236271

[20] Donner NC, Lowry CA. Sex differences in anxiety and emotional behavior. Pflugers Arch. 2013;465:601–26. doi: 10.1007/s00424-013-1271-7 23588380 PMC3805826

[21] Bharmal M, Fofana F, Dias Barbosa C, Williams P, Mahnke L, Marrel A, et al. Psychometric properties of the FACT-M questionnaire in patients with Merkel cell carcinoma. Health Qual Life Outcomes. 2017;15:247. doi: 10.1186/s12955-017-0815-5 29273043 PMC5741938

[22] Askew RL, Swartz RJ, Xing Y, Cantor SB, Ross MI, Gershenwald JE, et al. Mapping FACT-melanoma quality-of-life scores to EQ-5D health utility weights. Value Health. 2011; 14:900–6. doi: 10.1016/j.jval.2011.04.003 21914512

[23] Costello AB, Osborne JW. Best Practices in Exploratory Factor Analysis: Four Recommendations for Getting the Most From Your Analysis. Pract Assess Res Evaluat 2005; 10:1–9.

[24] Gagne P, Hancock GR. Measurement Model Quality, Sample Size, and Solution Propriety in Confirmatory Factor Models. Multivariate Behav Res 2006;41(1):65–83. doi: 10.1207/s15327906mbr4101_5 26788895

