# Supplementary figures and images for: Functional assessment of cancer therapy questionnaire for melanoma in the Serbian population: A factor analytic approach
Source: PLoS One. 2021 Jun 30;16(6):e0253937. doi: 10.1371/journal.pone.0253937 (PMC8244891; doi:10.1371/journal.pone.0253937)

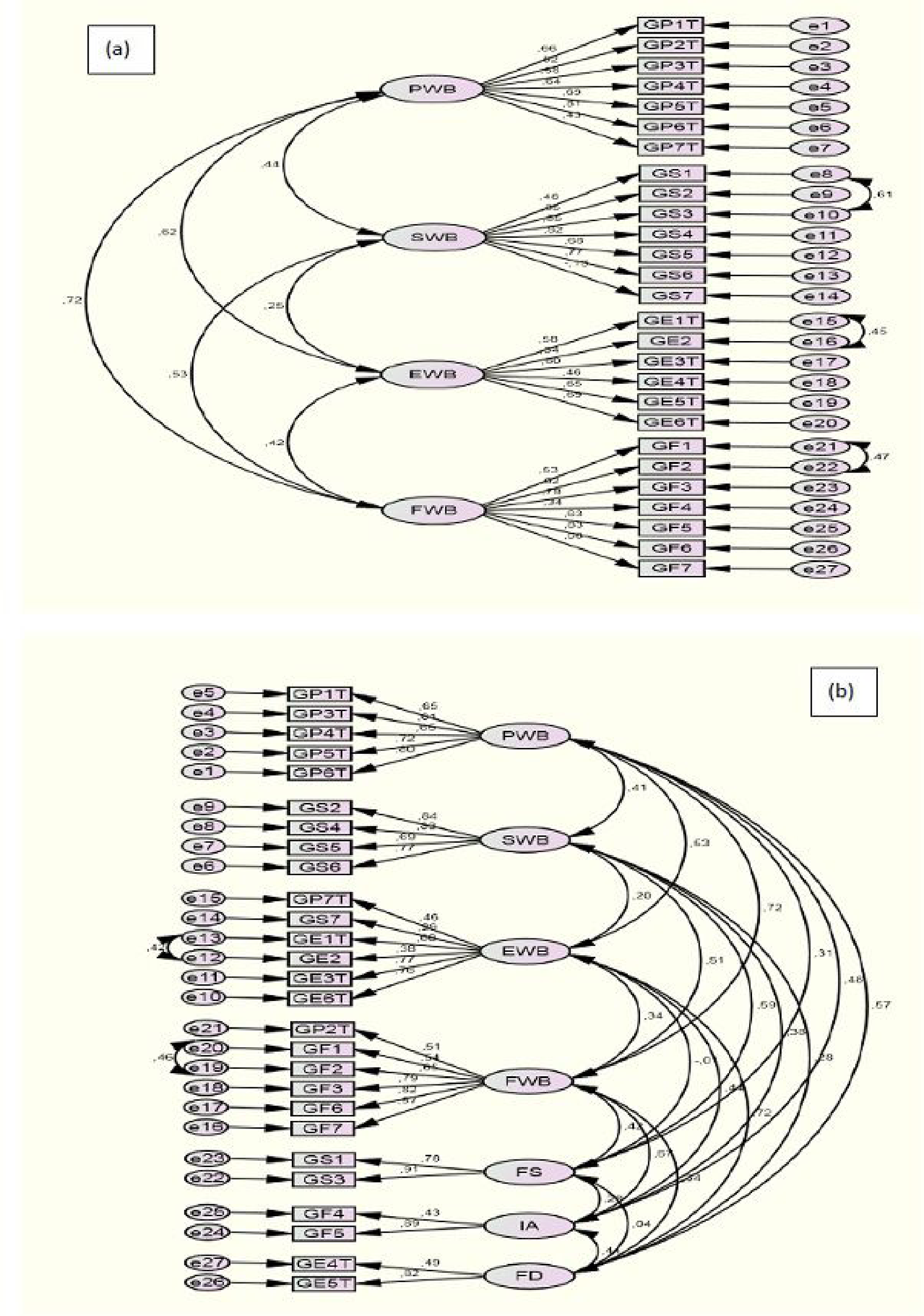

Supplement: S1 Fig — (TIF) [file pone.0253937.s001.tif]

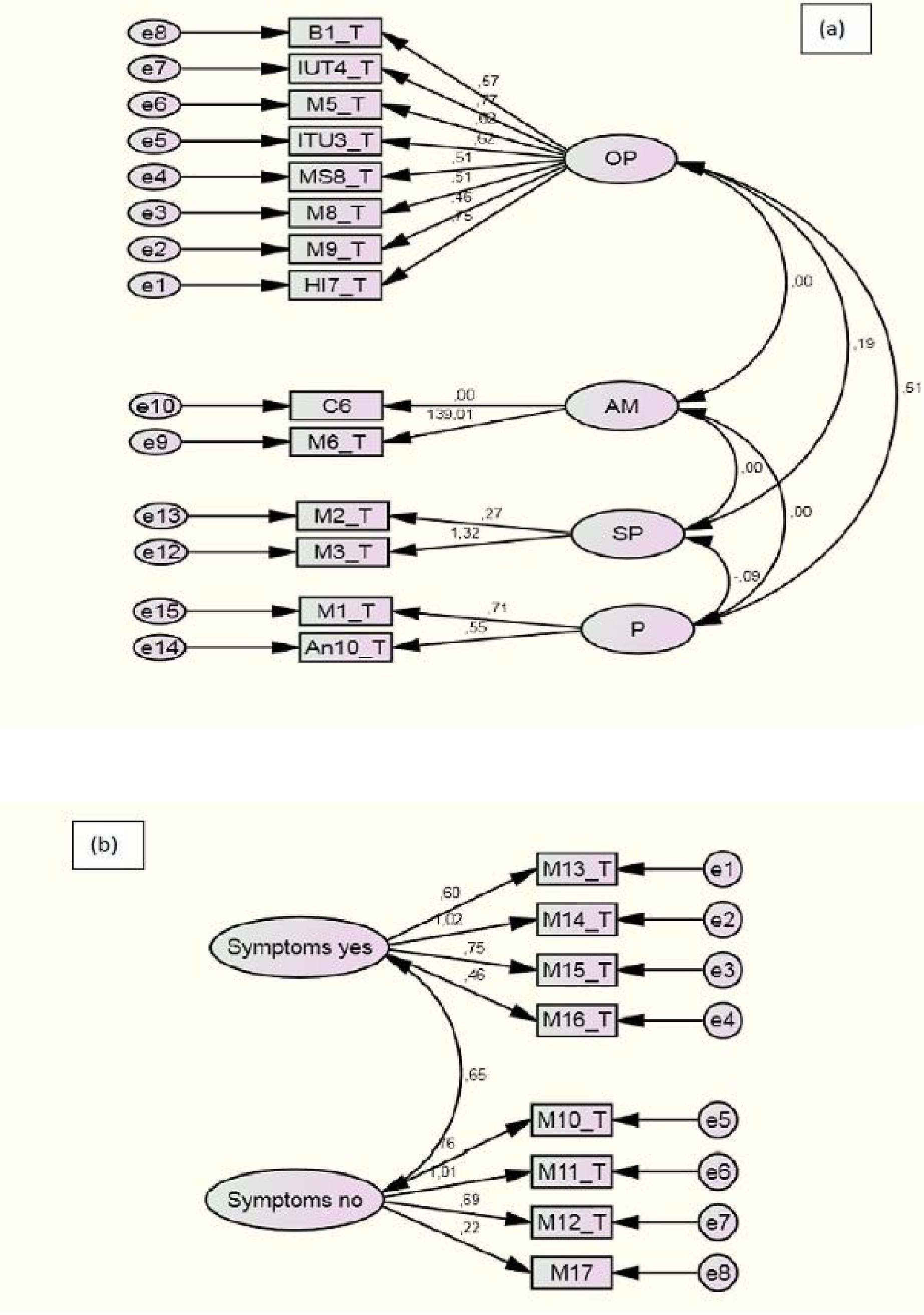

Supplement: S2 Fig — (TIF) [file pone.0253937.s002.tif]
