# Supplementary material for: Functional assessment of cancer therapy questionnaire for melanoma in the Serbian population: A factor analytic approach
Source: PLoS One. 2021 Jun 30;16(6):e0253937. doi: 10.1371/journal.pone.0253937 (PMC8244891; doi:10.1371/journal.pone.0253937)
Supplement: S1 Table — (DOC) [file pone.0253937.s003.doc]

**S1 Table**. Inter-subscale correlations

| **Subscale** | **PWB** | **SWB** | **EWB** | **FWB** | **MS** | **MSS** |
| --- | --- | --- | --- | --- | --- | --- |
| PWB | 1.00 |  |  |  |  |  |
| SWB | 0.303** | 1.00 |  |  |  |  |
| EWB | 0.546** | 0.329** | 1.00 |  |  |  |
| FWB | 0.524** | 0.456** | 0.429** | 1.00 |  |  |
| MS | 0.677** | 0.348** | 0.571** | 0.574** | 1.00 |  |
| MSS | 0.471** | 0.236* | 0.115 | 0.320** | 0.417** | 1.00 |

*p<0.05; **p<0.01; PWB – Physical wellbeing; SWB – Social/family wellbeing; EWB – Emotional wellbeing; FWB – Functional wellbeing; MS – Melanoma subscale; MSS – Melanoma surgery scale
