# Supplementary material for: Functional assessment of cancer therapy questionnaire for melanoma in the Serbian population: A factor analytic approach
Source: PLoS One. 2021 Jun 30;16(6):e0253937. doi: 10.1371/journal.pone.0253937 (PMC8244891; doi:10.1371/journal.pone.0253937)
Supplement: S2 Table — (DOC) [file pone.0253937.s004.doc]

**S2 Table**. Correlations of FACT-M domain scores with SF-36 questionnaire scores

| **Sub**  **scale** | **PF** | **RP** | **RE** | **VT** | **MH** | **SF** | **P** | **GH** | **PCS** | **MCS** | **Total** |
| --- | --- | --- | --- | --- | --- | --- | --- | --- | --- | --- | --- |
| **PWB** | 0.544** | 0.564** | 0.521** | 0.622** | 0.508** | 0.590** | 0.549** | 0.409** | 0.634** | 0.674** | 0.694** |
| **SWB** | 0.295** | 0.282* | 0.218 | 0.327** | 0.205 | 0.225* | 0.250* | 0.151 | 0.303** | 0.282* | 0.296** |
| **EWB** | 0.179 | 0.325** | 0.359** | 0.324** | 0.514** | 0.398** | 0.317** | 0.307** | 0.334** | 0.451** | 0.405** |
| **FWB** | 0.591** | 0.497** | 0.512** | 0.610** | 0.399** | 0.515** | 0.414** | 0.553** | 0.606** | 0.627** | 0.637** |
| **MS** | 0.468** | 0.437** | 0.475** | 0.512** | 0.451** | 0.501** | 0.514** | 0.593** | 0.582** | 0.586** | 0.613** |
| **MSS** | 0.385** | 0.370** | 0.204 | 0.311** | 0.160 | 0.287** | 0.354** | 0.191 | 0.412** | 0.284* | 0.376** |
| **FACT-M Total** | 0.541** | 0.539** | 0.508** | 0.596** | 0.484** | 0.558** | 0.505** | 0.534** | 0.631** | 0.643** | 0.667** |

*p<0.05; **p<0.01; PWB – Physical well being; SWB – Social/family well being; EWB – Emotional well being; FWB – Functional well being; MS – Melanoma subscale; MSS – Melanoma surgery scale; PF - Physical functioning; RP – Role limitations due to physical health; RE – Role limitations due to emotional problems; VT - Vitality; MH – Mental health; SF – Social functioning; P - Pain; GH – General health; PCS – Physical composite score; MCS – Mental composite score
