# Supplementary material for: Functional assessment of cancer therapy questionnaire for melanoma in the Serbian population: A factor analytic approach
Source: PLoS One. 2021 Jun 30;16(6):e0253937. doi: 10.1371/journal.pone.0253937 (PMC8244891; doi:10.1371/journal.pone.0253937)
Supplement: S1 Appendix — (DOC) [file pone.0253937.s005.doc]

**FACT-M Questionnaire**

Below is a list of statements that other people with your illness have said are important**. Please circle or mark one number per line to indicate your response as it applies to the past 7 days.**

**Not**

**A little**

**Some-**

**Quite**

**Very**

|  | **PPHYSICAL WELL-BEING** | **at all** | **bit** | **what** | **a bit** | **much** |
| --- | --- | --- | --- | --- | --- | --- |
| GP1 | I have a lack of energy...................................................... | 0 | 1 | 2 | 3 | 4 |
| GP2 | I have nausea .................................................................... | 0 | 1 | 2 | 3 | 4 |
| GP3 | Because of my physical condition, I have trouble  meeting the needs of my family ........................................ | 0 | 1 | 2 | 3 | 4 |
| GP4 | I have pain........................................................................ | 0 | 1 | 2 | 3 | 4 |
| GP5 | I am bothered by side effects of treatment......................... | 0 | 1 | 2 | 3 | 4 |
| GP6 | I feel ill............................................................................. | 0 | 1 | 2 | 3 | 4 |
| GP7 | I am forced to spend time in bed ....................................... | 0 | 1 | 2 | 3 | 4 |
| **SOCIAL/FAMILY WELL-BEING Not** | | | **A little** | **Some-** | **Quite** | **Very** |
|  |  | **at all** | **bit** | **what** | **a bit** | **much** |
| GS1 | I feel close to my friends................................................... | 0 | 1 | 2 | 3 | 4 |
| GS2 | I get emotional support from my family............................ | 0 | 1 | 2 | 3 | 4 |
| GS3 | I get support from my friends............................................ | 0 | 1 | 2 | 3 | 4 |
| GS4 | My family has accepted my illness ................................... | 0 | 1 | 2 | 3 | 4 |
| GS5 | I am satisfied with family communication about my illness ............................................................................... | 0 | 1 | 2 | 3 | 4 |
| GS6 | I feel close to my partner (or the person who is my main support) ............................................................................ | 0 | 1 | 2 | 3 | 4 |
| Q1 | *Regardless of your current level of sexual activity, please answer the following question. If you prefer not to answer it,* |  |  |  |  |  |
|  | *please mark this box and go to the next section.* |  |  |  |  |  |
| GS7 | I am satisfied with my sex life .......................................... | 0 | 1 | 2 | 3 | 4 |

Please circle or mark one number per line to indicate your response as it applies to the past 7 days.

| **EMOTIONAL WELL-BEING Not** | | | **A little** | **Some-** | **Quite** | **Very** |
| --- | --- | --- | --- | --- | --- | --- |
|  |  | **at all** | **bit** | **what** | **a bit** | **much** |
| GE1 | I feel sad........................................................................... | 0 | 1 | 2 | 3 | 4 |
| GE2 | I am satisfied with how I am coping with my illness ......... | 0 | 1 | 2 | 3 | 4 |
| GE3 | I am losing hope in the fight against my illness................. | 0 | 1 | 2 | 3 | 4 |
| GE4 | I feel nervous.................................................................... | 0 | 1 | 2 | 3 | 4 |
| GE5 | I worry about dying .......................................................... | 0 | 1 | 2 | 3 | 4 |
| GE6 | I worry that my condition will get worse........................... | 0 | 1 | 2 | 3 | 4 |
| **FUNCTIONAL WELL-BEING Not** | | | **A little** | **Some-** | **Quite** | **Very** |
|  |  | **at all** | **bit** | **what** | **a bit** | **much** |
| GF1 | I am able to work (include work at home)......................... | 0 | 1 | 2 | 3 | 4 |
| GF2 | My work (include work at home) is fulfilling.................... | 0 | 1 | 2 | 3 | 4 |
| GF3 | I am able to enjoy life ....................................................... | 0 | 1 | 2 | 3 | 4 |
| GF4 | I have accepted my illness ................................................ | 0 | 1 | 2 | 3 | 4 |
| GF5 | I am sleeping well............................................................. | 0 | 1 | 2 | 3 | 4 |
| GF6 | I am enjoying the things I usually do for fun..................... | 0 | 1 | 2 | 3 | 4 |
| GF7 | I am content with the quality of my life right now............. | 0 | 1 | 2 | 3 | 4 |

Please circle or mark one number per line to indicate your response as it applies to the past 7 days.

| **ADDITIONAL CONCERNS Not at** | | | **A little** | **Some-** | **Quite** | **Very** |
| --- | --- | --- | --- | --- | --- | --- |
|  |  | **all** | **bit** | **what** | **a bit** | **much** |
| M1 | I have pain at my melanoma site or surgical site ............... | 0 | 1 | 2 | 3 | 4 |
| M2 | I have noticed new changes in my skin (lumps, bumps, color(colour)) ................................................................... | 0 | 1 | 2 | 3 | 4 |
| M3 | I worry about the appearance of surgical scars .................. | 0 | 1 | 2 | 3 | 4 |
| B1 | I have been short of breath................................................ | 0 | 1 | 2 | 3 | 4 |
| ITU4 | I have to limit my physical activity because of my condition .......................................................................... | 0 | 1 | 2 | 3 | 4 |
| An10 | I get headaches ................................................................. | 0 | 1 | 2 | 3 | 4 |
| Hep3 | I have had fevers (episodes of high body temperature)...... | 0 | 1 | 2 | 3 | 4 |
| C1 | I have swelling or cramps in my stomach area .................. | 0 | 1 | 2 | 3 | 4 |
| C6 | I have a good appetite....................................................... | 0 | 1 | 2 | 3 | 4 |
| M5 | I have aches and pains in my bones .................................. | 0 | 1 | 2 | 3 | 4 |
| M6 | I have noticed blood in my stool ....................................... | 0 | 1 | 2 | 3 | 4 |
| ITU3 | I have to limit my social activity because of my  condition .......................................................................... | 0 | 1 | 2 | 3 | 4 |
| MS8 | I feel overwhelmed by my condition................................. | 0 | 1 | 2 | 3 | 4 |
| M8 | I isolate myself from others because of my condition........ | 0 | 1 | 2 | 3 | 4 |
| M9 | I have difficulty thinking clearly (remembering, concentrating)................................................................... | 0 | 1 | 2 | 3 | 4 |
| HI7 | I feel fatigued ................................................................... | 0 | 1 | 2 | 3 | 4 |

Please circle or mark one number per line to indicate your response as it applies to the past 7 days.

| ***At the site of my melanoma surgery:* Not** | | | **A little** | **Some-** | **Quite** | **Very** |
| --- | --- | --- | --- | --- | --- | --- |
|  |  | **at all** | **bit** | **what** | **a bit** | **much** |
| M10 | I have swelling at my melanoma site ................................ | 0 | 1 | 2 | 3 | 4 |
| M11 | I have swelling as a result of surgery ................................ | 0 | 1 | 2 | 3 | 4 |
| M12 | I am bothered by the amount of swelling .......................... | 0 | 1 | 2 | 3 | 4 |
| M13 | Movement of my swollen area is painful .......................... | 0 | 1 | 2 | 3 | 4 |
| M14 | Swelling keeps me from doing the things I want to do ...... | 0 | 1 | 2 | 3 | 4 |
| M15 | Swelling keeps me from wearing clothes or shoes I want to wear ............................................................................. | 0 | 1 | 2 | 3 | 4 |
| M16 | I feel numbness at my surgical site ................................... | 0 | 1 | 2 | 3 | 4 |
| M17 | I have good range of movement in my arm or leg ............. | 0 | 1 | 2 | 3 | 4 |
